# In Vitro Modulation of Gut Microbiota and Metabolism by Cooked Cowpea and Black Bean

**DOI:** 10.3390/foods9070861

**Published:** 2020-07-01

**Authors:** Catarina Teixeira-Guedes, Tereza Sánchez-Moya, Cristina Pereira-Wilson, Gaspar Ros-Berruezo, Rubén López-Nicolás

**Affiliations:** 1 Department of Biology, University of Minho, 4710-057 Braga, Portugal; cigteixeira@gmail.com (C.T.-G.); cpereira.bio.uminho@gmail.com (C.P.-W.); 2 Centre for the Research and Technology of Agro-Environmental and Biological Sciences, University of Trás-os-Montes and Alto Douro, 5000-801 Vila Real, Portugal; 3 Department of Food Science and Human Nutrition, Faculty of Veterinary Sciences, Regional Campus of International Excellence Campus Mare Nostrum, University of Murcia, 30100 Murcia, Spain; teresasm@um.es (T.S.-M.); gros@um.es (G.R.-B.)

**Keywords:** cowpea, black bean, in vitro digestion, in vitro fermentation, prebiotic effect, short-chain fatty acids

## Abstract

Legumes are a rich source of a wide range of compounds that may represent an important tool to overcome gut dysbiosis. In this work, the prebiotic potential of two cooked legumes (cowpea and black bean) was investigated in comparison with potato:beef mixture, as substrates in batch faecal culture fermentation. Prior to the fermentation, all the samples were in vitro digested, passing through three phases, namely mouth, gastric and small intestine simulation, and then in vitro fermented for 6, 24 and 48 h. The shift of pH, production of gas and short-chain fatty acids (SCFAs) and changes in gut microbiota were evaluated along the fermentation time. The pH decreased significantly over time in all media with fermentable sources when compared with the negative control. Gas production was higher in the media containing fermentable source than in the negative control and decreased with fermentation time. The concentration of SCFAs increased over time and it was significantly higher for both legumes than in inulin (positive control) and potato:beef meal. Acetate was the major SCFAs produced during fermentation, particularly in media containing legumes. Both legumes presented a strong prebiotic effect on gut microbiota, showing a significant increase in *Bifidobacterium* and *Lactobacillus*. These results suggest that consumption of cooked cowpea and black bean, used alone or as an ingredient of novel functional foods, may contribute to improving intestinal health and therefore human health promotion.

## 1. Introduction 

Native microbiota is an essential component of the modern concept of human health and disease. It has been shown that the gut microbiota plays an important role in basic human biological processes, such as modulating the metabolic phenotype, regulating the epithelial development and influencing the innate immune system [1,2]. Furthermore, a microbiota dysbiosis has been associated with developments of chronic diseases, such as obesity, diabetes, metabolic syndrome, non-alcoholic fatty liver disease, cirrhosis, hepatocarcinoma and inflammatory bowel disease, among others [2]. The microbiota has the capacity of increasing the energy extraction from food by increasing nutrient acquisition, metabolism of undigested carbohydrates and vitamin biosynthesis [3], as well as playing an important role in satiety [4]. In addition, microbiota also provides a physical barrier to pathogens [5,6,7] and it is essential to the development of intestinal mucosa and the immune system [2,8,9]. It has been established that dietary interventions may lead to microbial modifications, suggesting that diet could be used for the improvement of the overall health of individuals exhibiting dysbiosis and associated diseases [10].

Dietary components are capable of modulating gut microbiota through effects on proliferation and/or stimulation of metabolic activity of some bacterial populations [11]. In this context, prebiotics are important components of healthy diets and represent a useful dietary approach supporting healthy large intestine microbiota [12]. The definition of prebiotics has undergone subsequent changes over the years and it is currently defined as “a non-viable food component that confers a health benefit on the host associated with modulation of the microbiota” [13]. Prebiotics resist the digestive enzymes in the human gut and enter the large intestine without suffering modification to their structure. There, they are fermented by the gut microbiota producing substances such as short-chain fatty acids (SCFAs) (mainly acetate, propionate and butyrate), L-lactate, CO_2_ and H_2_, and stimulating the growth of lactic acid bacteria and bifidobacteria, among others [14]. SCFAs act as energetic substrates for the host, accounting for up to 10% of the daily dietary energy or used in other metabolic processes. They can also decrease gut pH and may inhibit gastrointestinal pathogens [15]. Microbial-synthesized SCFAs contribute 70% of ATP production in the colon, with butyrate as the preferred fuel for colonocytes [10,16].

It has been recently demonstrated that legumes or pulses could be used as an important tool to overcome gut dysbiosis [14,17]. They are an excellent source of protein, vitamins (folate), minerals (iron) and antioxidants, being low in fat [18,19,20]. Carbohydrates are the major component in beans, accounting from 55% to 75% of the dry weight. Most of carbohydrates are starches, consisting of amylose (linear chain) amylopectin (branched chain) [21,22]. Compared with other carbohydrate– rich foods (cereals and potatoes), it has a higher amylose:amylopectin ratio, being correlated with higher resistant starch content. Resistant starch (soluble and insoluble fiber), as well as non-digestive oligosaccharides, may resist small intestine hydrolysis but can be fermented in the large intestine by the colonic microbiota [23]. Furthermore, they present a considerable amount of dietary fiber (14% to 19%) and oligosaccharides. More than 50% of fibers are insoluble, being composed of pectins, pentosans, hemicellulose, cellulose and lignin [22]. In addition, legumes contain several bioactive substances including enzyme inhibitors, lectins, phytates and phenolic compounds. These polyphenolic compounds consist mainly of tannins, phenolic acids and flavonoids that can be metabolized by the gut microbiota, affecting intestinal health [24,25,26]. 

In the last years, a large number of studies have been performed on the effect of purified oligosaccharides on the gut microbiota composition [16]. However, the effect of food sources rich in prebiotic components is scarce, especially regarding legumes, and even less in the way we consume it—cooked. Based on this, we propose to study the impact of two cooked legumes (cowpea and black bean) and traditional food (potato and beef) on the pH shift, intestinal microbiota and the metabolism of SCFA using in vitro human batch fermentation. 

## 2. Materials and Methods 

### 2.1. Sample Preparation 

Cowpea seeds (*Vigna unguiculata*) and back bean (*Phaseolus vulgaris*) were obtained from a local distributor in Portugal. Beans were soaked overnight in distilled water and then cooked until getting a smooth texture. Potato and beef were also cooked. The cooking water was removed and the samples were milled. In order to perform a comparison, a mixture of potatoes and beef was performed to equate the content of carbohydrates and protein present in legumes under study. The composition in carbohydrates, protein and water corresponded to approximately 18%, 9% and 67%, respectively.

### 2.2. Static In Vitro Simulation of Gastrointestinal Food Digestion

The simulated gastrointestinal digestion was performed according to the protocol previously described by Minekus et al. [27] and Sánchez-Moya et al. [28] and consisted of the simulation of three phases: oral, gastric and intestinal digestion. The simulated salivary fluid (SSF), simulated gastric fluid (SGF) and simulated intestinal fluid (SIF) were prepared according to Minekus et al. [27] The oral phase started by mixing the food ingredient (5 g) with 5 mL of SSF containing 150 UmL^−1^ α-amylase (final concentration of 75 UmL^−1^). The mixture was homogenized and maintained for 2 min in a water bath shaker (60 strokes per minute) at 37 °C. In the gastric phase, the SGF was added to the oral phase at a final ratio of 50:50 (v:v). Pepsin (Sigma–Aldrich, St Louis, MO, USA) was added to a final concentration of 2000 UmL^−1^. The pH of the gastric phase was adjusted to 3 with 1 M of HCl and placed in the water bath shaker for 2 h in the same conditions of the oral phase. Finally, to simulate the intestinal phase, SIF was added to the gastric phase to a final volume of 50:50 (v:v). In this phase, the bile salts at a final concentration of 10 mM (Sigma–Aldrich) and pancreatin at a final concentration of 100 UmL^−1^ (Sigma–Aldrich) were added. The pH was adjusted at 7 using 6 M of NaOH and the mixture was placed under the same conditions for 2 h. At the end of the digestion process, samples were stopped on ice and frozen at −80 °C until performing the batch culture fermentation.

### 2.3. Fecal Sample Preparation

The samples of human microbiota were obtained from 3 healthy volunteers (non-smokers, non-pregnant and did not take antibiotics, pro and prebiotics in the last 3 months). In addition, the donors presented stable and normal weight in the last 3 months and had no clinical history of metabolic and intestinal diseases. All experiments were performed according to the laws and institutional ethics guidelines. The Ethical Research Committee of the University of Murcia approved this experiment (ID: 1964/2018).

The minimal basal medium (MBM) was prepared according to Sánchez-Moya et al. [28] and González-Bermúdez et al. [29].

The fermentation was performed in digested cowpea, black bean, potato:beef and inulin (prebiotic positive control) as substrates. Wheaton serum bottles were prepared with MBM and substrate at the final concentration of 1% (w:v) and placed in anaerobic conditions.

Fecal samples were collected in the morning, placed into sterile disposable containers under anaerobic conditions and processed within 30 min after the collection in order to maintain the original composition in an optimal environment [30]. The faeces from the three volunteers were mixed in pre-reduced phosphate buffer at a final concentration of solid/solvent of 1:9 (w/v) and then homogenized in a stomacher. After that, the homogenized was inoculated in Wheaton bottles with pre-reduced MBM at a final concentration of 10%. The inoculated media was kept for 6 h at 37 °C in a water bath shaker (60 strokes per minute) to stabilize. Digested samples and controls were inoculated with 10% of stabilized microbiota and maintained under anaerobic conditions for 48 h. Every sample was performed in quadruplicate. The pH and gas production were recorded at 0, 6, 24 and 48 h of incubation and samples were collected for SCFA determination and quantification of bacteria. 

### 2.4. DNA Extraction and Microbiota Analysis from Fermented Samples

All material used in DNA extraction were autoclaved twice and were RNase-free. The DNA extraction was performed according to the method previously described by Boon et al. [8]. Quantitative real-time PCR (qPCR) of 16S rRNA gene-targeted group-specific primers were used to characterize the changes in the microbiota [28]. The qPCR was performed in quadruplicate according to Sánchez-Moya et al. [28] and González-Bermúdez et al. [29]. The reaction was performed in a final volume of 25 µL, containing 1 µL of DNA template, 12.5 µL of SensiMixTM SYBR No-ROX (Bioline, London, UK), 0.5 µL of forward and reverse primer (0.2 µM) (Table 1) and 10.5 µL of nuclease-free water (AppliChem, Darmstadt, Germany). The qPCR was performed in 96-well CFX96 real-time thermocycler and detection system (BioRad, Madrid, Spain). The program was set at 95 °C for 10 min for DNA denaturation and enzyme activation, 40 cycles of denaturation (95 °C for 15 s), annealing (60 °C for 30 s) and extension (72 °C for 45 s). The melting curve was obtained from 65 °C to 95 °C, increasing 0.5 °C at each 0.5 s. The concentration of each bacteria was calculated based on cycle threshold (Ct) values and the standard curve obtained from serial 10-fold dilution of pure culture DNA corresponding to 10^8^ to 10^2^ cells equivalent per mL. Changes in bacterial populations were expressed as logarithm of genome equivalent per mL.

### 2.5. SCFA Quantification by Gas Chromatography

The SCFA analysis was performed in a gas chromatograph system (7890A Agilent technologies, Santa Clara, USA) with a flame ionization detector and a NukolTM GC-column (30 m × 0.25 × 0.25 µm) according to the method described by Sánchez-Moya et al. [28] and González-Bermúdez et al. [29]. Results were analyzed using Agilent Chemstation Operation Software (Santa Clara, USA). All samples were run in quadruplicate and results were expressed in mM. 

### 2.6. pH and Gas Production Assessment

At the time of aliquots collections (0, 6, 24 and 48 h) pH and gas increment production were determined by using pH-meter (Crison, Berlin, Germany) and a pressure transmitter (CCPT6200, WIKA Instruments, S.A.U., Barcelona, Spain), respectively [15,28,31]. After pH and gas determinations, 8 mL of each sample were removed and centrifuged (1200× *g*, 15 min). Supernatant and pellet were separately stored at −80 °C for measurement of SCFA production and microbiota quantification, respectively.

### 2.7. Statistical Analysis

Statistical analyses were performed using IBM SPSS statistics 21.0 software (SPSS Inc., Chicago, IL, USA). Differences between samples were tested using analysis of variance (ANOVA) followed by Tukey’s multiple comparisons tests. A *p* < 0.05 was considered as statistically significant. All determinations were conducted in quadruplicate and the results were presented as mean ± standard deviation (SD). 

## 3. Results

This study aims to investigate the prebiotic effects of cooked cowpea and black bean in comparison with a traditional food (potato:beef) on gut microbiota and metabolites. The food samples were in vitro digested followed by batch culture fermentation with microbial cultures isolated from fecal samples of healthy donors. The production of SCFAs (acetate, propionate and butyrate), the pH shift, gas generation and changes in the microbial population were determined. Inulin, which has been recognized as a prebiotic gold standard, was used as positive control and medium without carbon source as the negative control.

### 3.1. Changes in Bacteria Population in Fecal Batch Culture

The quantitative changes in the in vitro culture of the human fecal bacteria populations, expressed in log genome equivalents per ml, are summarized in Table 2. In the negative control, *Bifidobacterium* and *Lactobacillus* did not show significant variation over fermentation time (*p* > 0.05), while *Enterobacteriaceae*, *Firmicutes* and *Bacteroides* displayed a slight increase after 6 h incubation followed by a decrease at 24 and 48 h (*p* < 0.05). For both legumes and inulin, a significant increase was observed in *Bifidobacterium*, *Lactobacillus*, *Enterobacteriaceae* and *Firmicutes* until 24 h fermentation and in *Bacteroides* only until 6 h followed by a significant decrease (*p* < 0.05). In media containing potato:beef, for all groups of bacteria studied (*Bifidobacterium*, *Lactobacillus*, *Enterobacteriaceae*, *Firmicutes* and *Bacteroides*) the increase was observed only until 6 h of fermentation. 

Considering the effects of fermentation on *Bifidobacterium*, we found higher increase for media containing cowpea and black bean (1.46-fold and 1.39-fold, respectively) than potato: beef or inulin (1.33-fold and 1.31-fold, respectively), when compared to the negative control. The *Bifidobacterium* shift was significantly higher for cowpea and black bean than for inulin at 24 h fermentation, showing the potential bifidogenic effect of these foods. 

In the *Lactobacillus* group, the medium containing cowpea and black beans increased the bacterial population after 6 h of fermentation and maintained until 24 h (*p* < 0.05). Media containing potato:beef increased significantly the *Lactobacillus* population at 6 h of fermentation and inulin at 24 h followed by a decrease. The highest increase in bacterial population was found for media containing inulin and cowpea at 24 h fermentation (2.79-fold and 2.51-fold, respectively) when compared to the negative control. *Enterobacteriaceae* increased in all carbon sources after 6 h fermentation followed by a reduction after 24 h. *Bacteroides*, which comprises approximately 30% of total colonic microbiota, increased significantly after 6 h of fermentation in all media, including the negative control and decreased after 24 h. In *Firmicutes*, cowpea and inulin showed an increase in the bacterial population until 24 h, whereas the traditional food only until 6 h.

### 3.2. Production of SCFA, Gas and pH Shift 

**Figure 1:**
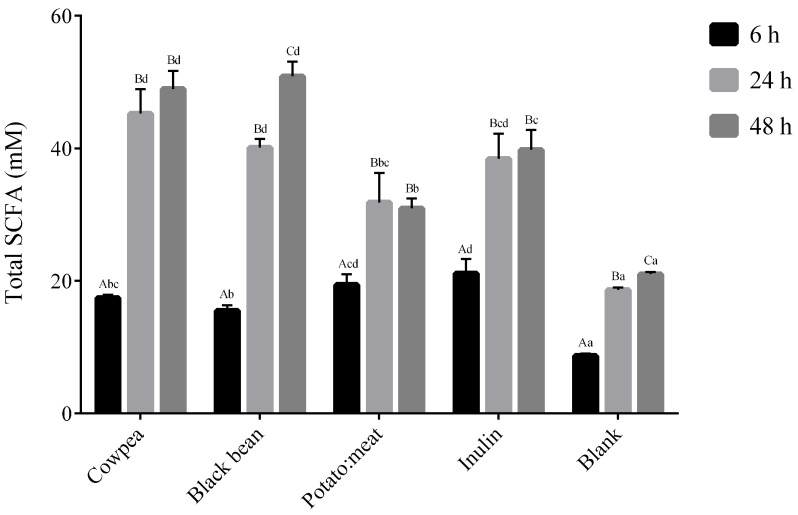
Total short-chains fatty acids (acetate, propionate and butyrate) produced during fermentation time (6, 24 and 48 h). Results are expressed in mM. The data marked by the same letters were not significantly different (*p* > 0.05). Different uppercase letters for comparison within each fermentable source at a different time and lowercase letters for comparison within the different fermentable source at the same time, indicating significant differences at *p* < 0.05.

The total production of SCFAs (acetate, propionate and butyrate) along the fermentation time is presented in Figure 1. The concentration (expressed in mM) of total SCFAs increased significantly during fermentation time for all samples. As expected, the negative control (or blank) presented the lowest values of SCFAs. At 6 h of fermentation, potato: beef and inulin displayed a significant increase of analyzed fatty acids when compared to both legumes. At 24 and 48 h of fermentation, a switch in the positions, cowpea and black bean showing the highest values of SCFAs (approximately 50 mM) (*p* < 0.05). No significant differences were observed between the legumes.

The molar proportion of the main fatty acid, acetate, propionate and butyrate are presented in Table 3. Acetate was the most abundant SCFA found in all substrates, reaching the highest concentration at 48 h fermentation for both cowpea and black bean samples (approximately 42 mM). Regarding propionate, the highest values were detected after 48 h of fermentation of cowpea and black bean (around 5.9 mM) and after 24 h in potato:beef and inulin (6.0 and 5.3 mM, respectively). Values of butyrate increased over fermentation time for all carbon sources, inulin showed the highest values especially at 24 h incubation.

**Figure 2:**
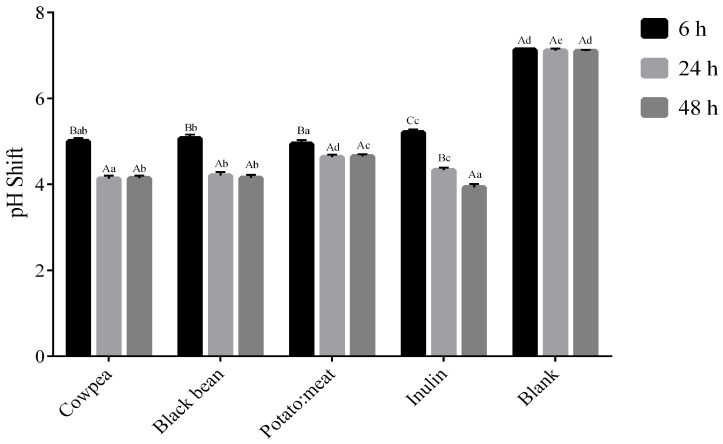
pH shift during fermentation time (6, 24 and 48 h). Results presented as mean and standard deviation (*n* = 3). The data marked by the same letters were not significantly different (*p* > 0.05). Different uppercase letters for comparison within each fermentable source at a different time and lowercase letters for comparison within the different fermentable source at the same time, indicating significant differences at *p* < 0.05.

Concerning the pH, samples with carbon sources presented significantly lower values when compared to the blank. pH values decreased significantly during the fermentation time in all samples, except in the blank (Figure 2). A statistically significant decrease (*p* < 0.05) at 6 h and 24 h was observed in all samples and the pH remained stable reaching similar values at 48 h. However, in the case of inulin this decrease continued until the end of the experiment, obtaining the lowest pH value of all tested samples (*p* < 0.05). It is also worthy of note that potato:beef sample reached the lowest pH at 6 h (4.9) (*p* < 0.05), but this value was maintained along the fermentation time (4.6).

**Figure 3:**
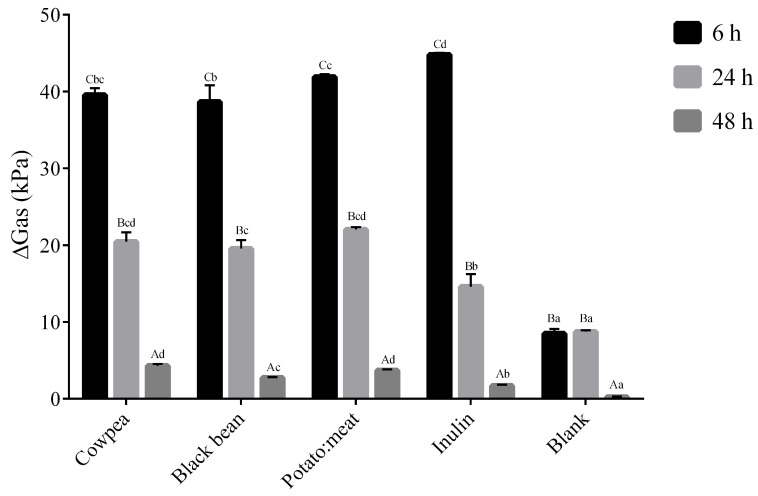
Gas increment during fermentation time (kPa). Results are expressed as mean and standard deviation. The data marked by the same letters were not significantly different (*p* > 0.05). Different uppercase letters for comparison within each fermentable source at a different time and lowercase letter for comparison within the different fermentable source at the same time, indicating significant differences at *p* < 0.05.

Regarding the increment in gas production (Figure 3), the highest values were recorded at 6 h of fermentation in all samples when compared to the negative control (*p* < 0.05), decreasing at the subsequent times. The highest gas increment was observed at 6 h for inulin (*p* < 0.05). At the subsequent times, increments in gas production were lower than the rest of the samples tested (*p* < 0.05).

## 4. Discussion

In recent years, a new health paradigm has evolved, placing more emphasis on the beneficial aspects of the diet. Although the primary role of the diet is to provide nutrients to fulfil metabolic requirements, the use of foods to improve health and wellbeing is being increasingly accepted [32]. It has been demonstrated that ingestion of certain dietary components is associated with potentially beneficial bacterial groups, with enterotypes becoming established by long-term eating habits [33]. 

Prebiotic carbohydrates are a specific colonic nutrient, comprising oligosaccharides and complex carbohydrates, which act as biosynthetic precursors for human microbiota providing subtracts for their metabolic activity. These prebiotic carbohydrates need to resist digestive processes in the upper gastrointestinal tract being fermented by intestinal microbiota, thereby selectively stimulating the growth and activity of health-promoting bacteria [34].

Although the prebiotic effect is mostly attributed to fermentable carbohydrates, protein can also be fermented in the large intestine and have a prebiotic effect [28]. The digestion of proteins and subsequent absorption in the upper intestine is not fully efficient and about 10% can reach the large intestine and become available for fermentation by the colonic microbiota [35].

In this perspective, our study intended to characterize the prebiotic effects of two cooked legumes (cowpea and black bean) in comparison with a traditional meal (potato:beef). For this, an in vitro model of digestion and fermentation were applied, and the pH shift, production of gas and SCFA were recorded and changes in the gut microbiota evaluated using qPCR. 

The in vitro fermentation models are considered excellent tools that allow the screening of many substances, ranging from food samples, dietary ingredients and even drugs, and assess how they alter and are altered by the gastrointestinal environments and microbial populations. These models provide the first assessment of the types of metabolites formed and the effect on gut microbial populations. Although valuable, this method has limitations that preclude direct inferences of in vivo effects, namely the substrate depletion and the accumulation of the end-products of microbial metabolism that alter the conditions in the batch away from the balanced starting point [36,37].

Nowadays, another tool is being applied for the study of the fecal microbiota, namely the next-generation sequencing (NGS). However, it also has some disadvantages, such as the relative abundance quantification, difficulty of statistical treatments, cost and so on. Moreover, the library preparation of this NGS causes a slight estimation of Bacteroides to compare to qPCR [38]. On the other hand, qPCR provides some interesting features such as cost-effectiveness and feasibility, simplicity in lab-working, as well as statistical treatment [38].

Regarding our results, relatively to specific microbial groups, both legumes demonstrated certain selectivity to *Bifidobacterium*. The increase in *Bifidobacterium* was similar in media containing cowpea or black bean and significant differences were found in comparison with inulin and potato:beef. This result confirms the suitability of these subtracts as fermentable sources to *Bifidobacterium* growth and, therefore, their bifidogenic (prebiotic) effect. To our knowledge, no studies have investigated the in vitro bifidogenic effect of cooked cowpea and black bean. However, this effect has already been reported for flour from uncooked lupin and broad bean [14] and lentils:chickpeas (50:50) [11]. The in vitro bifidogenic effect was also reported in colonic fermentation with purified GOS, FOS and inulin [15,16,39]. Despite the fact that the species that are responsible for the maintenance of the eubiosis in the human gut microbiota are not fully clarified, *Bifidobacterium* is widely considered to play an important role in host health and wellbeing. 

The favorable shifts in SCFA profiles found in the present study in cultures using fecal inoculum should, however, not be attributed only to the bifidogenic effect, but also to the change of other microbial groups.

The *Lactobacillus* group showed significant growth with all carbon sources tested, registering an increase of 2.50-fold for legumes and 2.80-fold for inulin. This is an important observation since a significant increase in this microbial group was observed for both legumes. This finding is in agreement, but still much higher than that previously reported by Gullón et al. [14] for lupin, broad bean (1.14-fold) and FOS (1.10-fold). Raffinose-derived oligosaccharides have also been reported to increase *Lactobacillus* population [40] whereas in another study, GOS did not induce significant differences [39]. 

In the *Bacteroides–Prevotella*, which is the predominant genus within the lower human intestinal tract, a similar increase was observed at 6 h of incubation with all fermentable sources and blank followed by a decreased at 24 and 48 h. Interestingly, this result is not in accordance with that reported in uncooked broad beans flour and FOS [14] and lentil: chickpea flour (50:50) [11], where a slight increase in this bacterial population was observed. It is known that *Bacteroides* are strongly influenced by pH, growing optimally at approximate pH 6.5–7 and poorly at pH 5.5 [28,41,42]. Our data showed a significant decrease in pH after 6 h fermentation, which might explain the small differences between fermentable sources and blank. The pH values decreased even more after 24 h and 48 h of fermentation, which may have induced the marked decreased in *Bacteroides* growths. 

The composition of the gut microbiota is influenced by both the type and overall amount of dietary substrate (carbohydrates, protein and fat) in the diet. In addition, changes in the bacterial composition affect many microbial metabolic processes, including the production of SCFAs, gas (CO_2_, CH_4_ and H_2_) and pH shift [16,39]. Moreover, these parameters may also have feedback affecting the bacterial composition. 

The most abundant end-products of microbial metabolism detectable after batch fermentation are acetate, propionate and butyrate. SCFAs are mainly produced in the proximal colon in high concentrations and transported through the distal colon by the intestinal flow. These bacterial metabolites have been associated with multiple biological activities in the host, such as the regulation of energy homeostasis, anti-inflammatory activity and satiety, among others [16,28].

The impact of each of the three main fermentation products differs, but these weak acids all act to lower colonic pH, thereby preventing the growth of other potentially pathogenic bacteria [16]. The changes observed in the total SCFA and the SCFA profile agreed with the microbial population shown in the present study. As expected, both the increase in SCFA amount and the decrease in pH along fermentation time were considerably more pronounced in the presence of fermentable sources, especially legumes and inulin, when compared to the negative control cultures. 

Gas production is an inevitable product of microbial fermentation in anaerobic ecosystems and an indicator of the activities of the total gut microflora. However, gas formation is not a universal trait among anaerobic bacteria and the biochemistry of some species involves no gas generation at all. This is the case for common probiotics like *Lactobacillus* and *Bifidobacterium* [43]. In agreement with this, in our study, high gas production and lower *Bifidobacterium* and *Lactobacillus* were found at the beginning of fermentation time. 

Acetate was clearly the most prevalent SCFA produced with all the substrates tested and it reached the highest concentrations in media containing both legumes after 48 h of fermentation. The predominant formation of acetate agrees with the results reported in fecal fermentation of purified GOS [39], lupin and broad bean flours [14], lentils:chickpeas (50:50) flour [11] and non-digestible fraction of common bean [44]. Although it is difficult to establish a relationship between the production of short-chain fatty acids with specific bacteria, especially in vitro tests, the exponential increase in acetate seems to correlate with the exponential increment in *Bifidobacterium* and *Lactobacillus*. 

Regarding propionic and butyric acid concentrations, the highest values were detected after 48 h of fermentation of legumes in comparison with inulin. Propionate plays an important role in Krebs cycle and acts as a gluconeogenesis precursor. The effects in hepatic carbohydrate metabolism are supported by its role in improving glucose tolerance and insulin sensitivity, as well as increasing high-density lipoprotein (HDL) [16,32].

Butyrate is the main energy source for colonocytes that constitute the epithelial lining of the large intestine. The increase of butyrate production by gut bacteria has been related to the maintenance of colonic epithelium formation and protection against colorectal cancer [14,16]. In our experiment, an overall increase of butyrate was observed along fermentation time, especially for the media containing inulin (more than 1.5-fold increased). *Firmicutes* are recognized as one of the main groups of butyrate-producing bacteria [28,45] and are accordingly increased in this fermentable source compared to the others.

## 5. Conclusions

The present work supports the prebiotic effects of two cooked legumes, cowpea and black bean, using in vitro fecal batch microbiota from healthy donors when compared to a traditional meal (potato:beef). Both legumes stimulate the growth of probiotic bacteria, demonstrated by the highest increase in *Lactobacillus* and *Bifidobacterium* when compared to the traditional meal (potato:beef) and inulin. In addition, both legumes increased the total SCFA significantly, especially acetate, when compared with the other fermentable sources. This suggests that legumes alone or used as ingredients may constitute a good source of prebiotic components that are relevant to gastrointestinal health and could be used for functional foods and/or nutraceuticals to overcome gut dysbiosis.

## Figures and Tables

**Table 1:** 16S rRNA gene-targeted group-specific primers used in this study according to Sánchez-Moya et al. [28] and González-Bermúdez et al. [29].

Bacteria	Primers
***Bifidobacterium***	Fwd: GATTCTGGCTCAGGATGAACGC
	Rv: CTGATAGGACGCGACCCCAT
***Lactobacillus***	Fwd: AGCAGTAGGGAATCTTCCA
	Rv: CATGGAGTTCCACTGTCCTC
***Enterobacteriaceae***	Fwd: TGCCGTAACTTCGGGAGAAGGCA
	Rv: TCAAGGACCAGTGTTCAGTGTC
***Firmicutes***	Fwd: GGAGYATGTGGTTTAATTCGAAGCA
	Rv: AGCTGACGACAACCATGCAC
***Bacteroides–Prevotella***	Fwd: GAGAGGAAGGTCCCCCAC
	Rv: CGKACTTGGCTGGTTCAG

**Table 2:** Bacterial population of fecal batch cultures at 0, 6, 24 and 48 h of fermentation using different fermentable sources.

	*Time (h)*	*Bifidobacterium*	*Lactobacillus*	*Enterobacteriaceae*	*Firmicutes*	*Bacteroides*
***Cowpea***	0	5.34 ± 0.05^Aa^	1.48 ± 0.13^Aa^	4.19 ± 0.03^Aa^	5.38 ± 0.09^Aa^	5.81 ± 0.10^Ba^
	6	6.73 ± 0.11^Bbc^	3.74 ± 0.14^Cb^	5.04 ± 0.04^Cb^	5.90 ± 0.10^BCab^	6.04 ± 0.09^Ba^
	24	7.55 ± 0.24^Dd^	3.77 ± 0.17^Cb^	4.95 ± 0.07^Cc^	6.02 ± 0.13^Cc^	4.87 ± 0.20^Ab^
	48	7.20 ± 0.16^Cd^	3.40 ± 0.12^Bb^	4.67 ± 0.07^Bd^	5.70 ± 0.11^Bbc^	4.61 ± 0.21^Ab^
***Black bean***	0	5.34 ± 0.05^Aa^	1.48 ± 0.13^Aa^	4.19 ± 0.03^Aa^	5.38 ± 0.09^Aa^	5.81 ± 0.10^Ba^
	6	6.67 ± 0.21^Bbc^	3.53 ± 0.29^Bb^	4.99 ± 0.13^Cb^	5.78 ± 0.29^Cab^	6.06 ± 0.31^Ba^
	24	7.31 ± 0.04^Ccd^	3.56 ± 0.02^Bb^	4.69 ± 0.08^Bb^	5.75 ± 0.03^BCbc^	4.71 ± 0.02^Ab^
	48	6.89 ± 0.13^Bc^	3.43 ± 0.09^Bb^	4.35 ± 0.17^Ac^	5.42 ± 0.11^ABab^	4.66 ± 0.17^Ab^
***Potato:beef***	0	5.34 ± 0.05^Aa^	1.48 ± 0.13^Aa^	4.19 ± 0.03^Aa^	5.38 ± 0.09^Aa^	5.81 ± 0.10^Ba^
	6	7.10 ± 0.18^Cc^	4.20 ± 0.27^Cb^	5.36 ± 0.17^Bc^	6.28 ± 0.20^Bb^	6.23 ± 0.20^Ba^
	24	6.63 ± 0.12^Bb^	3.43 ± 0.25^Bb^	5.20 ± 0.06^Bc^	5.55 ± 0.25^Ab^	3.84 ± 0.14^Aa^
	48	6.58 ± 0.08^Bb^	3.84 ± 0.06^BCc^	5.11 ± 0.21^Be^	5.37 ± 0.16^Aa^	3.53 ± 0.49^Aa^
***Inulin***	0	5.34 ± 0.05^Aa^	1.48 ± 0.13^Aa^	4.19 ± 0.03^Aa^	5.38 ± 0.09^Aa^	5.81 ± 0.10^ABa^
	6	6.76 ± 0.52^BCbc^	2.45 ± 0.61^Ba^	4.89 ± 0.22^Bb^	6.06 ± 0.44^BCab^	6.18 ± 0.53^Ba^
	24	7.05 ± 0.11^Cc^	4.19 ± 0.11^Cc^	4.72 ± 0.15^Bb^	6.50 ± 0.15^Cd^	5.97 ± 0.15^ABd^
	48	6.46 ± 0.11^Bb^	3.83 ± 0.22^Cc^	3.96 ± 0.17^Aab^	5.92 ± 0.13^Bc^	5.39 ± 0.12^Ac^
***Blank***	0	5.34 ± 0.05^Aa^	1.48 ± 0.13^Aa^	4.19 ± 0.03^Ba^	5.38 ± 0.09^Aa^	5.81 ± 0.10^Aa^
	6	5.54 ± 0.26^Aa^	1.76 ± 0.22^Aa^	4.84 ± 0.19^Cb^	5.65 ± 0.20^Ba^	6.23 ± 0.25^Ba^
	24	5.26 ± 0.12^Aa^	1.50 ± 0.20^Aa^	4.27 ± 0.04^Ba^	5.18 ± 0.02^Aa^	5.64 ± 0.05^Ac^
	48	4.93 ± 0.07^Aa^	1.63 ± 0.09^Aa^	3.72 ± 0.06^Aa^	5.19 ± 0.05^Aa^	5.52 ± 0.10^Ac^

Results are expressed as log genome equivalent per mL and presented as mean ± SD from four replicates (*n* = 4). The data marked by the same letters were not significantly different (*p* > 0.05). Different uppercase letters for comparison within each fermentable source at a different time and lowercase letters for comparison within the different fermentable source at the same time, indicating significant differences at *p* < 0.05.

**Table 3:** Production of short-chain fatty acids (SCFA) during the fermentation time of 6, 24 and 48 h.

	*Time (h)*	*Acetate (mM)*	*Propionate (mM)*	*Butyrate (mM)*
	6	11.79 ± 0.44^Abc^	4.48 ± 0.09^Ac^	1.17 ± 0.04^Aab^
**Cowpea**	24	38.09 ± 3.64^Bd^	5.67 ± 0.10^Bb^	1.45 ± 0.10^Aab^
	48	41.45 ± 2.92^Bd^	5.97 ± 0.46^Bb^	1.55 ± 0.31^Aa^
	6	10.28 ± 0.60^Ab^	4.18 ± 0.22^Ac^	1.04 ± 0.01^Aa^
**Black bean**	24	34.09 ± 3.64^Bcd^	5.67 ± 0.10^Bb^	1.35 ± 0.09^Ba^
	48	43.45 ± 2.20^Cd^	5.80 ± 0.03^Cb^	1.61 ± 0.02^Ba^
	6	13.13 ± 1.54^Acd^	4.85 ± 0.42^Ac^	1.45 ± 0.14^Abc^
**Potato:meat**	24	24.07 ± 3.22^Bb^	6.00 ± 0.89^Ab^	1.77 ± 0.34^Abc^
	48	23.14 ± 1.19^Ba^	5.89 ± 0.64^Ab^	1.91 ± 0.32^Aa^
	6	14.46 ± 1.59^Ad^	4.24 ± 0.40^Ac^	2.43 ± 0.18^Ad^
**Inulin**	24	29.19 ± 2.5^Bbc^	5.36 ± 0.37^Ba^	3.59 ± 0.20^Bd^
	48	30.83 ± 2.89^Bc^	5.24 ± 0.15^Bb^	3.69 ± 0.05^Bc^
	6	5.11 ± 0.03^Aa^	2.20 ± 0.27^Aa^	1.05 ± 0.10^Aa^
**Blank**	24	11.36 ± 0.55^Ba^	3.04 ± 0.02^Ba^	2.01 ± 0.04^Bc^
	48	12.57 ± 0.01^Ca^	3.66 ± 0.26^Ca^	2.49 ± 0.03^Cb^

Results of SCFA are expressed in mM. Data presented as mean ± SD from four replicates (*n* = 4). The data marked by the same letters were not significantly different (*p* > 0.05). Different uppercase letters for comparison within each fermentable source at a different time and lowercase letters for comparison within the different fermentable source at the same time, indicating significant differences at *p* < 0.05.
